# Behavioral and neural responses to social exclusion in women: the role of facial attractiveness and friendliness

**DOI:** 10.1038/s41598-024-65833-4

**Published:** 2024-07-02

**Authors:** Tracy Vaillancourt, Stefon van Noordt, Amanda Krygsman, Heather Brittain, Adam C. Davis, Iryna S. Palamarchuk, Steven Arnocky, Sidney J. Segalowitz, Michael J. Crowley, Louis A. Schmidt

**Affiliations:** 1 Counselling Psychology, Faculty of Education, University of Ottawa, 145 Jean-Jacques-Lussier, Ottawa, ON K1N 6N5 Canada; 2 Mount St. Vincent University, Halifax, Canada; 3 Canadore College, North Bay, Canada; 4 University of Toronto, Toronto, Canada; 5 Nipissing University, North Bay, Canada; 6 Brock University, St. Catharines, Canada; 7 Yale University, New Haven, United States; 8 McMaster University, Hamilton, Canada

**Keywords:** Experimental evolution, Sexual selection, Social evolution, Evolution

## Abstract

The behavioral and neural responses to social exclusion were examined in women randomized to four conditions, varying in levels of attractiveness and friendliness. Informed by evolutionary theory, we predicted that being socially excluded by attractive unfriendly women would be more distressing than being excluded by unattractive women, irrespective of their friendliness level. Our results contradicted most of our predictions but provide important insights into women’s responses to interpersonal conflict. Accounting for rejection sensitivity, P300 event-related potential amplitudes were largest when women were excluded by unattractive unfriendly women. This may be due to an expectancy violation or an annoyance with being excluded by women low on social desirability. An examination of anger rumination rates by condition suggests the latter. Only attractive women’s attractiveness ratings were lowered in the unfriendly condition, indicating they were specifically punished for their exclusionary behavior. Women were more likely to select attractive women to compete against with one exception—they selected the Black attractive opponent less often than the White attractive opponent when presented as unfriendly. Finally, consistent with studies on retaliation in relation to social exclusion, women tended to rate competitors who rejected them as being more rude, more competitive, less attractive, less nice, and less happy than non-competitors. The ubiquity of social exclusion and its pointed emotional and physiological impact on women demands more research on this topic.

## Introduction

The need to belong is a fundamental human motivator^1^. When this biologically driven need is unmet, children, adolescents, and adults suffer—they experience more loneliness, more internalizing and externalizing problems, and have poorer health^2^. In the past two decades, there has been increased attention paid to the behavioral and neural basis of belonging using a cognitive neuroscience framework^3^. In this study, we extend this research using an evolutionary informed approach to examine the effect of social exclusion *on women*.

### Evolutionary significance of belonging for women

The neural underpinnings of social pain, defined as experiences that “signal the loss, or potential loss, of social connection or social value, therefore signifying an increased survival risk”^4^^,p.421^, have provided support for the hypothesis that social pain serves an important adaptive function—it acts as a neural alarm for not belonging that is salient enough to motivate individuals to cooperate and fit in and thus survive^5^. In ancestral times, not belonging would have been particularly costly for women and their offspring because in every culture, and throughout history, women have had greater parental investment than men^6^, and because the survival of offspring has been inextricably linked to maternal survival^7,8^. Given these realities, support from other women was needed to help with child rearing (alloparenting networks^9,10^) and the accrual of physical and psychological benefits like food acquisition, protection, and mutual aid^11,12^. Accordingly, belonging has been important to women’s survival (and that of their offspring) over deep evolutionary time^13,14^. This is not to suggest that belonging has not mattered for the survival of men and their offspring. Cooperation among men would have benefited the acquisition of resources like territory, food, and mates^15^, including via coalitional warfare^16^. Nevertheless, ancestral labour divisions would have fostered “sexually asymmetric cultural values and expectations whereby different traits and affordances became differentially valuable in and to men and women, creating a feedback loop between culture and our evolved psychology”^17^^,p.982^. An example of this type of evolutionary process is how, under conditions of stress, women tend to adopt the tend-and-befriend strategy (protecting offspring and seeking out social support for defense) over the fight-or-flight strategy that men are more likely to employ^18,19^.

### The impact of social exclusion on women

Evidence of the strong need to belong for women is observed in modern interpersonal interactions. Women form closer, more intimate social bonds than men^20^, which makes the experience of social exclusion more salient and more harmful to them^21^. For example, although women are more willing to socially exclude others and report being excluded at higher rates than men, they also perceive cues of social exclusion more quickly and have a more pronounced physiological reaction (increased heart rate) to exclusion than men^22^. Women are also hurt more than men by cues of social exclusion^23^, and the negative impact of social exclusion on health and well-being is more problematic for women than for men^21^.

Social exclusion is a core feature of indirect aggression, entailing intention to harm others while masking the true injurious purpose^21^. Indirect aggression is used across the lifespan, with women and girls worldwide using and being the target of this form of aggression proportionately more than men and boys^24,25^. It has been proposed that indirect aggression is the behavioral manifestation of intrasexual competition^21^. Consistent with this hypothesis, the women who are most susceptible to being socially excluded by other women are those perceived to be physically attractive and/or sexually uninhibited^26–36^.

### Social status indicators in women

Attractive women are at risk of being targeted by other women because of intrasexual competition^21^, but attractiveness still benefits women in social and mating domains in relation to accessing resources and increased social status and influence^37–40^. For example, Buss et al.^17^ found that attractiveness was more closely linked to the social status of women than of men cross-culturally, and Krems et al.^41^ found that women’s physical attractiveness was used to infer status (for men, physical strength was used^42,43^). In another study, Haas and Gregory^42^ found that physical attractiveness was associated with higher social status and interactional power. Specifically, attractive women were able to influence others more than less attractive women, and this effect was stronger when the attractiveness levels between women dyads was more pronounced. Social status is a key component of hierarchies^44^ which are important from an evolutionary perspective in that they help individuals avoid costly disputes over resources^17^. Given the benefits of social order, it is not surprising that social status is also a fundamental human motivator found in men and women and across different age groups and cultures^45^. Social status confers prominence, prestige, and influence, which can affect the allocation of resources, mating behavior, mate value, access to allies and alloparental care, and the ability to resolve conflicts. For these reasons, high social status is associated with greater fitness than low social status^46,47^.

Because attractiveness gives women desired status^17,41^, it might be especially beneficial to be accepted and socially included by more physically attractive same-sex peers. Similarly attractive women should be more threatening than less attractive women because attractive women can more readily inflict costs such as debasing potential rivals. Indeed, attractive women have been shown to impose reputational sanctions more easily on others than less attractive women^41,48^. Fisher and Cox^48^ found that the derogation of a rival’s appearance was most successful when the gossiper was attractive; men only lowered their attractiveness estimation of a woman when she was disparaged by an attractive woman and not by an unattractive woman. In another more recent study, Krems et al.^41^ found that American and Indian women expected physically attractive women “to enact greater anger when thwarted by a same-sex other” than less attractive women.

Physical attractiveness is not the only robust indicator of social status in women—being unfriendly or cruel to others also plays a role. Several studies have demonstrated that high status adolescent girls are not only physically attractive, but they are also often mean^49–54^. According to Vaillancourt and Krems^55^, physical attractiveness affords girls social status which they maintain using indirect aggression. Although there are few studies on this phenomenon in adult women, some evidence suggests that attractive women are meaner than their less attractive peers. Sell et al.^56^ found that attractive women were more prone to anger, prevailed in conflicts, were more entitled, and reported greater utility of personal (and political) aggression than less attractive women. Bobadilla et al.^57^ found that for women, but not for men, facial attractiveness predicted derogating opponents, a common indirect aggression strategy^21^. Given that attractiveness and unfriendliness are markers of social status, it may be most hurtful for women to be excluded by attractive unfriendly (i.e., mean) women.

### Present study

In this study, we used a novel experimental setup where women were randomly grouped to experience exclusion by four different types of female pairs: (1) attractive and friendly, (2) attractive and unfriendly, (3) unattractive and friendly, and (4) unattractive and unfriendly. The aim was to investigate whether being excluded by women with certain characteristics “hurt” more. Social exclusion was provoked using Cyberball, a virtual computer ball-tossing game in which participants play against pretend players^58^. The experience of being excluded was assessed by having participants indicate the extent to which they experienced ostracism^59^ and by measuring their electroencephalography (EEG) changes that were time-locked to the exclusion events. Specifically, we measured the amplitude of the P300 (P3) event-related potential (ERP) component. The functional significance of amplitude shifts in the P3 are thought to reflect increased attention to a task event, typically when events are novel (often referred to as the P3a, anterior cortical regions) or when individuals detect task-relevant target stimuli (often referred to as the P3b, with more posterior cortical sources^60,61^. It is the latter subtype of P3 that we report here. The specific feature(s) that perturb attention allocation and increases in P3 responses vary across paradigms and broadly include events that are important, such stimuli that are novel, rare, infrequent, unexpected, as well as task-relevant targets^62–64^. Pertinent to the current study, P3 modulation has been observed to social stimuli, including the anticipation, experience, and reappraisal of exclusionary events^62,65–67^. In our paradigm, the relevant factors were only whether the participant was being treated fairly (from their point of view) and the face of the other players. Therefore, if the participants were indifferent to what happened, then the P3 amplitude responses would not differ across the trial types.

#### Predictions

Based on the evolutionary importance of belonging to women and on previous evidence demonstrating that physical attractiveness and unfriendliness are indicators of their social status, we predicted that women would favor playing against attractive unfriendly women the least and unattractive friendly women the most, and being socially excluded by attractive unfriendly women would elicit the strongest feelings of ostracism and the largest P3 response. Because social exclusion has been shown to elicit retaliation^68,69^, we also predicted that women would rate their competitors as being more rude, more competitive, less attractive, less nice, and less happy than their non-competitors.

As manipulation checks, we examined participants’ estimation of social exclusion (i.e., if the number of tosses received differed by condition) across the four conditions, and the extent to which they felt excluded and ignored. Given that all women were excluded (Methods), we did not expect to find differences across these indicators. As a sensitivity analysis, we considered the impact of individual differences in rejection sensitivity in relation to how participants reacted to being social excluded. Rejection sensitivity refers to an individual's inclination to anticipate and interpret rejection with anxiety or anger, in both obvious and uncertain circumstances^70,71^. Individuals higher on rejection sensitivity tend to monitor their interpersonal interactions for signs of rejection more than individuals lower on rejection sensitivity. This hypervigilance can affect their neural and physiological responses to stimuli that signal rejection^72–74^. In addition, and relevant to the present study, women tend to report higher rejection sensitivity than men^75^.

## Method

### Participants

Participants included 87 undergraduate women aged 18 to 22 (*M*_age_ = 19.31 years, *SD*_age_ = 1.11), recruited from the Integrated System of Participation in Research Student Pool at the University of Ottawa. Participants were primarily enrolled in the Faculty of Social Sciences (30.2%), the Faculty of Science (25.6%), the Faculty of Health Sciences (22.1%), and the Faculty of Arts (17.4%). Most participants were heterosexual (87.2%). The racial/ethnic composition of the sample was diverse: 41.9% were White, 16.3% Black, 12.8% South Asian, 7.0% Middle Eastern, 7.0% Asian, 2.3% Latina, and 12.8% Other. Participants were compensated with two research credit points, worth 2% of a course grade in psychology. Four participants were excluded from the analyses because of problems with their EEG recordings. The final sample used in the analyses below included 83 participants. Research ethics approval was obtained from the University of Ottawa Research Ethics Board. The research was conducted in accordance with the Government of Canada’s Tri-Council Policy Statement for Ethical Conduct for Research Involving Humans.

### Procedures

Participants were told that they would be taking part in a two-part study “examining how the brains of young adults react when they are competing against each other”. Written consent was first secured by all participants. Participants then completed an online questionnaire (Part 1; 15 min to complete) prior to coming to the laboratory. For the EEG portion of the study (Part 2; 60 min to complete), participants were randomly assigned to compete against women who were: (1) attractive friendly (AF; *n* = 21), (2) attractive unfriendly (AU; *n* = 21), (3) unattractive friendly (UF; *n* = 21), and (4) unattractive unfriendly (UU; *n* = 20).

Participants had their photograph taken when they arrived at the laboratory. To be consistent with the photographs of potential competitors in the four experimental conditions, their photograph was taken against a white backdrop with good lighting while wearing a grey cotton t-shirt. Participants were informed that their photograph would be uploaded onto our remote site and that they would be playing a computer game against women from other laboratories across Ontario, Canada. They were also told that, because they would eventually be rated by others on characteristics like attractiveness and friendliness, we needed all the participants at each site to wear the same t-shirt for the photograph so that the raters would not be influenced by clothing. This cover story was given so that the participants did not become suspicious about why their virtual opponents were all wearing a similar grey t-shirt.

**Figure 1:**
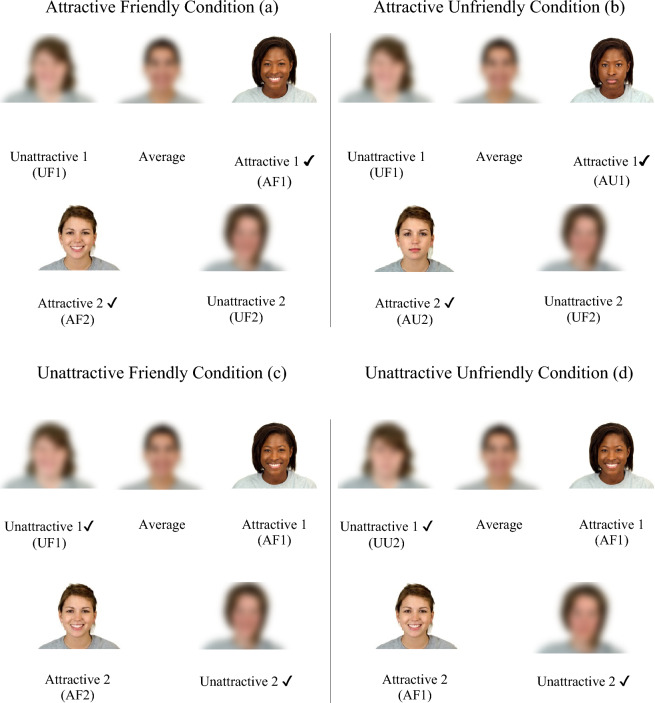
Landing page of potential opponents by condition. Note: Photographs of average and unattractive opponents are blurred due to the sensitive nature of the attractiveness ratings. Permission to use images was granted by the Chicago Face Database (CFD). CFD models provided written consent for their images to be used in publications. Procedures: Step 1: Participants in each condition are shown five possible opponents manipulated based on friendliness (**a**, **b**, **c**, or **d** depending on condition). Step 2: Participants selected two opponents to play against (**a**, **b**, **c**, or **d** depending on condition). Step 3: Cyberball game ostensibly “crashes” (blank screen presented). Step 4: New screen is populated with pictures of two opponents based on condition randomly assigned to (**a**, **b**, **c**, or **d** depending on condition; ✔ opponents played against), plus participant’s own picture (Fig. 2 for example of the *Attractive Friendly Condition*).

After the photograph was taken, participants were brought into another room to be fitted for the EEG sensor net. Once fitted, their photograph was uploaded onto the computer. Participants were then told that we would be connecting to the other laboratories. Once “connected”, participants were shown a screen of five competitors (images were of comparable size) who were currently available to play (Fig. 1). They were asked to pick two players who they wished to play against. The five potential competitors always included two unattractive White competitors (friendly (UF1 and UF2) or unfriendly (UU1 and UU2)), two attractive competitors (one Black and one White woman; friendly (AF1 and AF2) or unfriendly (AU1 and AU2)), and an average looking White woman who was smiling (average friendly (AverageF); see Fig. 1). Competitors’ images were taken from the Chicago Face Database and physical attractiveness ratings were based on 91 raters of the same race and gender^76^. In the friendly conditions, the potential opponents were smiling, and in the unfriendly conditions, they were not smiling. A neutral (i.e., not smiling) facial expression has been shown to be perceived as angry by women but not by men^31^. Neutral facial expressions are also perceived as less friendly and hostile by women than by men and this effect has been shown to be emotionally driven^77^. Participants rated the photographs at the end of the experiment and agreed with attractiveness ratings from the Chicago Face Database (“Results”).

**Figure 2:**
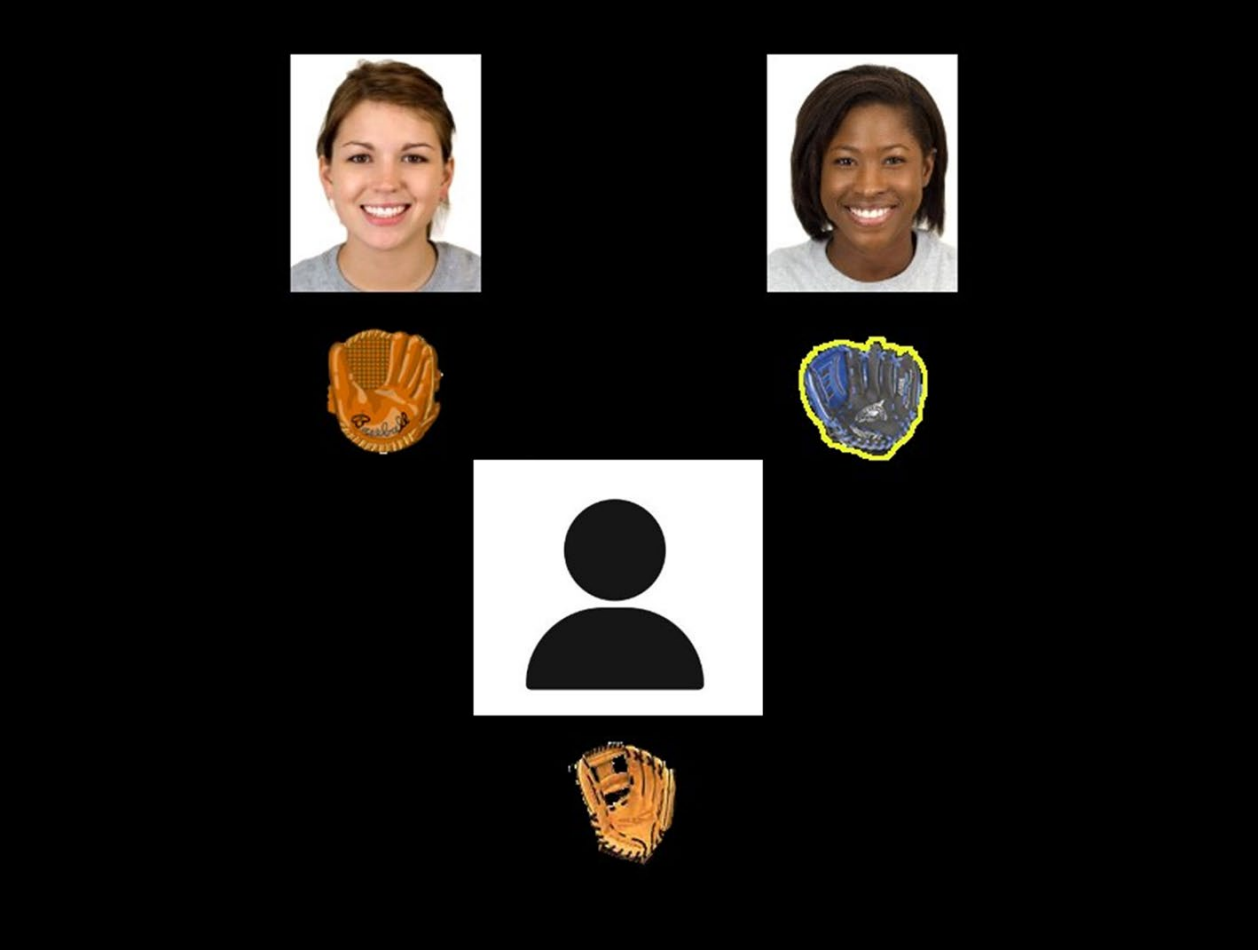
Attractive friendly condition. Note. Screenshot of Cyberball game with participant’s identity concealed. Yellow around glove indicates location of ball. Permission to use images was granted by the Chicago Face Database (CFD). CFD models provided written consent for their images to be used in publications.

After the selection of the opponents, the computer ostensibly “crashed”. This manipulation was done to help maintain the cover story (i.e., justifying why participants eventually only played against two opponents; see Fig. 2). As research assistants pretended to fix the issue, the screen re-appeared, but this time, only two opponents were available to compete against (AF, AU, UF, and UU). Participants were assured that this computer failure was fine because, in fact, only two additional participants were needed for the study. The Cyberball game was then played against the two “opponents”.

### Measures

#### Part 1: pre-EEG cyberball task

##### EEG screening

Participants were screened on potential EEG-related issues. Eligibility criteria for the EEG included that women were right-handed and had no self-reported history of brain injury or brain disease (e.g., concussion, brain cancer, epilepsy, Parkinson’s disease).

##### Rejection sensitivity

Rejection sensitivity was measured using the Adult Rejection Sensitivity Questionnaire^78,79^, an 18-item inventory that consists of 9 hypothetical situations describing interactions with partners, family, friends, and strangers (e.g., “You approach a close friend to talk after doing or saying something that seriously upset him/her.”). Participants were asked to assess their level of concern or anxiety and their anticipation of rejection using a 6-point Likert-type scale (1 = “very unconcerned,” to 6 = “very concerned,” and 1 = “very unlikely” to 6 = “very likely”). The internal consistency was good for the present study, α = 0.817.

#### Part 2: EEG cyberball task and post-EEG measures

##### Cyberball

The ERP version of Cyberball task^80^ was adapted to consist of a game in which a ball is tossed back and forth while participants were led to believe that they were interacting with two women from another Canadian university (Fig. 2). Participants used a response pad to throw to the left or right to their co-players displayed on the left or right, respectively. The co-players’ position (left or right) was computer-generated. The throws were pre-programmed so that participants could be thrown the ball (i.e., favor), participants throw the ball (i.e., ball-tosses), or the ball is passed between the virtual co-players (i.e., “not my turn” events). A fair play block had 108 trials (36 favors, 36 “not my turn” and 36 ball-tosses), followed by an exclusion block that consists of 47 trials (41 exclusion events, 3 favors, and 3 attention ball tosses, the latter being catch trials to assure that participants were paying attention to the task). Only data from the exclusion block were analyzed for this study. The final 36 exclusions were assessed and not the first five exclusions to ensure participants were aware they were being excluded. The exclusion phase of this task has been shown to be perceived as distressing to adults^58,81^.

##### Ostracism-induced need threat

At the end of the experiment, participants completed the Need Satisfaction Scale, a theory-derived measure assessing the psychological impact of social exclusion (i.e., ostracism;^59^). Three subscales (Belonging, 6 items; e.g., “I felt rejected.” reverse coded; Meaningful Existence, 5 items = e.g., “I felt invisible.” reverse coded), and Control, 5 items; e.g., “I felt I was unable to influence the action of others.” reverse coded) rated along a 5-point scale ranging from “not at all = 1” to “extremely or a lot = 5”, were combined into a single measure of ostracism-induced need threat. Higher scores indicating more need satisfaction, α = 0.877.

##### Rating of opponents

At the end of the experiment, coloured photographs of all 5 players who were originally offered as opponents were rated by the participants on how rude, competitive, attractive, happy, and nice they were on a 10-point scale ranging from “not at all descriptive = 1” to “extremely descriptive = 10”.

##### Manipulation check

Participants were asked to estimate how many throws they received in the last 40 throws, and they were asked to indicate the extent to which they were ignored and excluded (“not at all = 1” to “extremely or a lot = 5”; 59). They were also asked what they thought the purpose of the study was.

### EEG recording and pre-processing

A high-density EEG was recorded from 128 Ag/AgCl electrodes (Electrical Geodesics Incorporated [EGI], Inc.) with Netstation v.4.2 software (EGI, Inc.) and high impedance amplifiers, sampled at 250 Hz (0.1 Hz high pass, 100 Hz low pass) were used. All electrodes were referenced to Cz for recording and all impedances were at or under 40 kΩ before the Cyberball task was started. We used the E-prime v.2.0 (Psychology Software Tools, Inc.) software package to control our stimulus presentation.

A series of automated pre-processing procedures were conducted on the Shared Hierarchical Academic Research Computing Network (SHARCNet) in Octave 3.6.3, using the EEG Integrated Platform-Lossless^82^ pipeline (https://github.com/BUCANL/EEG-IP-L). This pipeline implements a standardized approach to assess the quality of signals through comprehensive data annotation of channel and independent component activity throughout the task recording, including identification of stereotypical artifacts such as eye-blinks, heart responses, muscle tension, and periods of relative non-stationarity. Specifically, the continuous EEG data were processed through a series of steps to assess measures of channel and independent component analysis (ICA) signal quality. For each assessment measure, the continuous data were epoched into 1-s non-overlapping time windows and the distribution values of interest (e.g., voltage variance) were examined against a criterion threshold (e.g., inter-quantile range) and data were then classified and flagged if they were consistent outliers (e.g., 20%). Following the automated procedures, a semi-automated interactive quality control was performed to review decisions on signal quality assessments. Figure 1S provides a schematic summary of the signal quality assessment measures, criterion threshold, outlier flagging, and the percentage of data that were identified as containing unreliable signals^82,83^. Overall, EEG recordings were stable such that, on average, 95.24% (*SD* = 3.87) of in-task time was retained and 80.98% (*SD* = 4.37) of channels were retained. Removed channels were spherical spline and all channels were re-referenced to the average.

### EEG post-processing and signal extraction

After quality control review, the remaining continuous data were segmented by task condition, time-locked to the onset of the exclusion feedback stimulus (− 200 to 800 ms) and baseline corrected using the 200 ms pre-stimulus period. Following the pre-processing there was a similar number of trials across the four groups (AF *M* = 31.67, *SD* = 4.64; UF *M* = 32.81, *SD* = 4.16; AU *M* = 31.3, *SD* = 4.59; UU *M* = 31.15, *SD* = 3.03). Like previous studies using the Cyberball paradigm^67,84,85^, the P3 was maximal at a central-posterior midline channel cluster and was averaged as a region-of-interest. The P3 was scored for each participant as the maximum amplitude in the 200–500 ms window following exclusion feedback (Fig. 1S).

## Analytic plan

The McNemar test of proportions was used to examine if different competitors were chosen at different frequencies. Conditions AF and UF were merged as all competitors were presented as friendly before starting the game. We compared how often each competitor was selected under three conditions—all friendly (AF/UF conditions), attractive unfriendly (AU), and unattractive unfriendly. A general linear model was used to examine if ostracism-induced need threat differed by condition and rejection-sensitivity. A robust statistical approach was used to group differences in P3 amplitude, which included percentile bootstrap tests using 20% trimmed means. Specifically, peak amplitudes in each group were re-sampled, with replacement, 1000 times. For each iteration, the mean was calculated after trimming the top and bottom 20% of the distribution. Significant effects in paired contrasts and interaction were assessed against the 95% confidence intervals in the distribution differences. To follow up, we examined group differences in the residual P3 amplitude after removing variance associated with individual differences in rejection sensitivity. Ratings of competitors’ attributes were assessed using a series of repeated measures ANOVAs. Where the assumption of sphericity was violated, the Greenhouse–Geisser‐corrected values are reported. Pairwise comparisons were examined and tested using Fisher’s least significant difference test to explore significant interactions. For all attributes, we examined differences between competitors within condition. For attractiveness, we also examined differences by condition within competitor. The Benjamini–Hochberg (BH) procedure for false discovery was applied^86^. Sensitivity analysis, using repeated measures ANCOVAs, was performed to examine if the results held when controlling for rejection sensitivity. Finally, one-way ANOVAs were used to examine whether the estimated number of tosses received, feeling ignored, or feeling excluded differed by condition. See Fig. 2S for analytic plan flowchart.

## Results

### Competitor selection

When all competitors were presented as friendly (*n* = 42), the attractive women were selected more often than unattractive women and the neutral woman (AF1 = 64.3%; AF2 = 66.7%; NF = 31.0%; UF1 = 21.4%; UF2 = 16.7%), Zs > |1.96|, with no differences between other competitors. When the attractive competitors were presented as unfriendly (*n* = 21), AU2 was selected more frequently than all other competitors (AU1 = 42.9%; AU2 = 76.2%; NF = 38.1%; UF1 = 28.6%; UF2 = 14.3%), Zs > |1.96|, whose proportions were equal. When the unattractive competitors were presented as unfriendly (*n* = 20), the attractive women were selected more often than unattractive unfriendly women and NF (AF1 = 75.0%; AF2 = 60.0%; NF = 35.0%; UU1 = 10.0%; UU2 = 20.0%), Zs > |1.96|, with no differences between other competitors.

### Ostracism-induced need threat

There was no interaction, *F*(3,74) = 1.978, *p* = 0.125, no main effect of condition, *F*(3,74) = 2.311, *p* = 0.083, and no main effect of rejection sensitivity, *F*(1,74) = 2.853, *p* = 0.095*.*

### P3 amplitude

**Figure 3:**
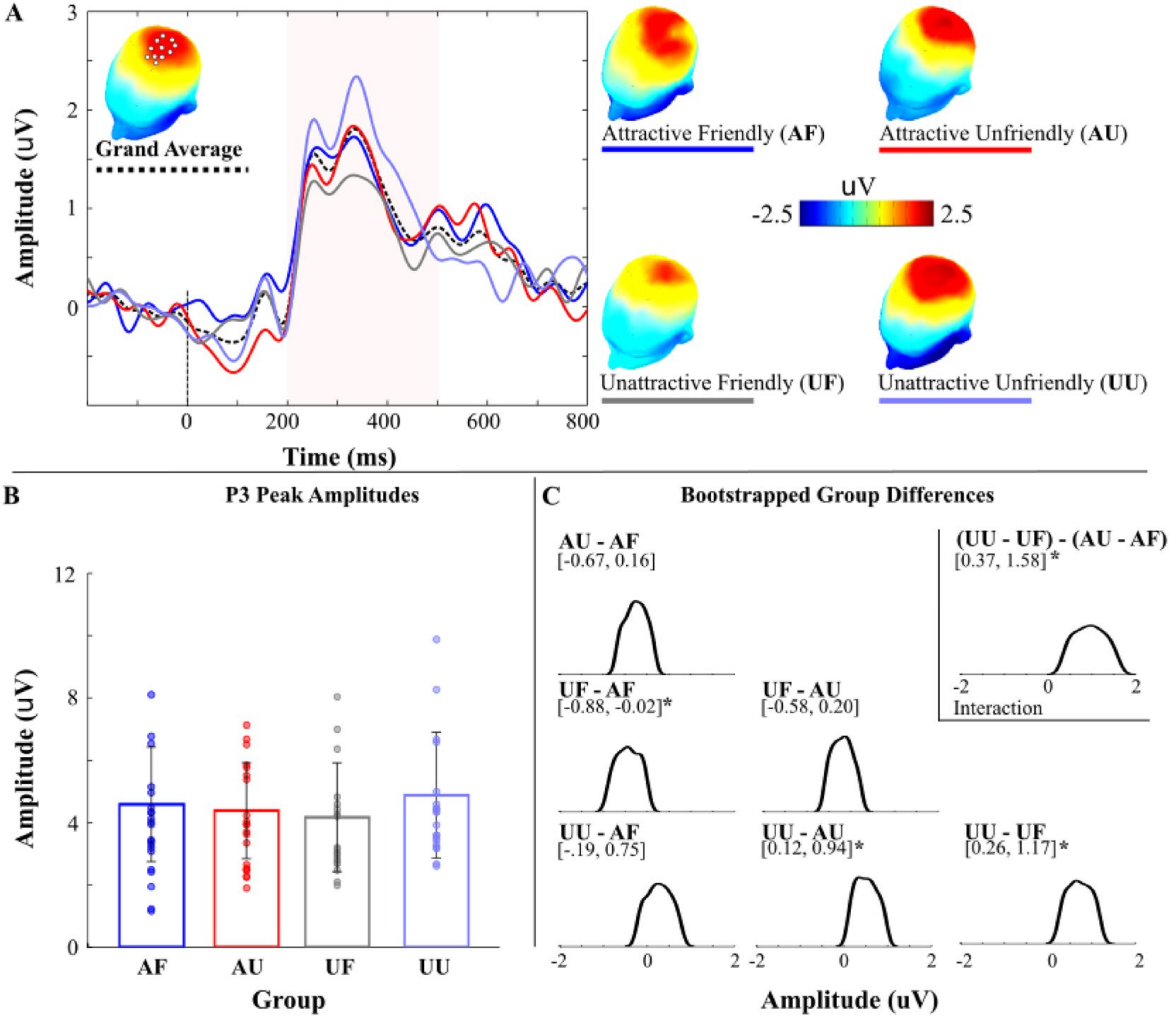
P3 Amplitude. (**A**) Group and grand average waveforms from central-posterior midline channel cluster (indicated by white dots on grand average map) and topographical maps. (**B**) Individual peak and group average peak P3 amplitudes. Error bars are ± 1 *SD*. Panel (**C**) Distribution plots of group bootstrapped trimmed means and paired comparison differences. *p < 0.05.

**Figure 4:**
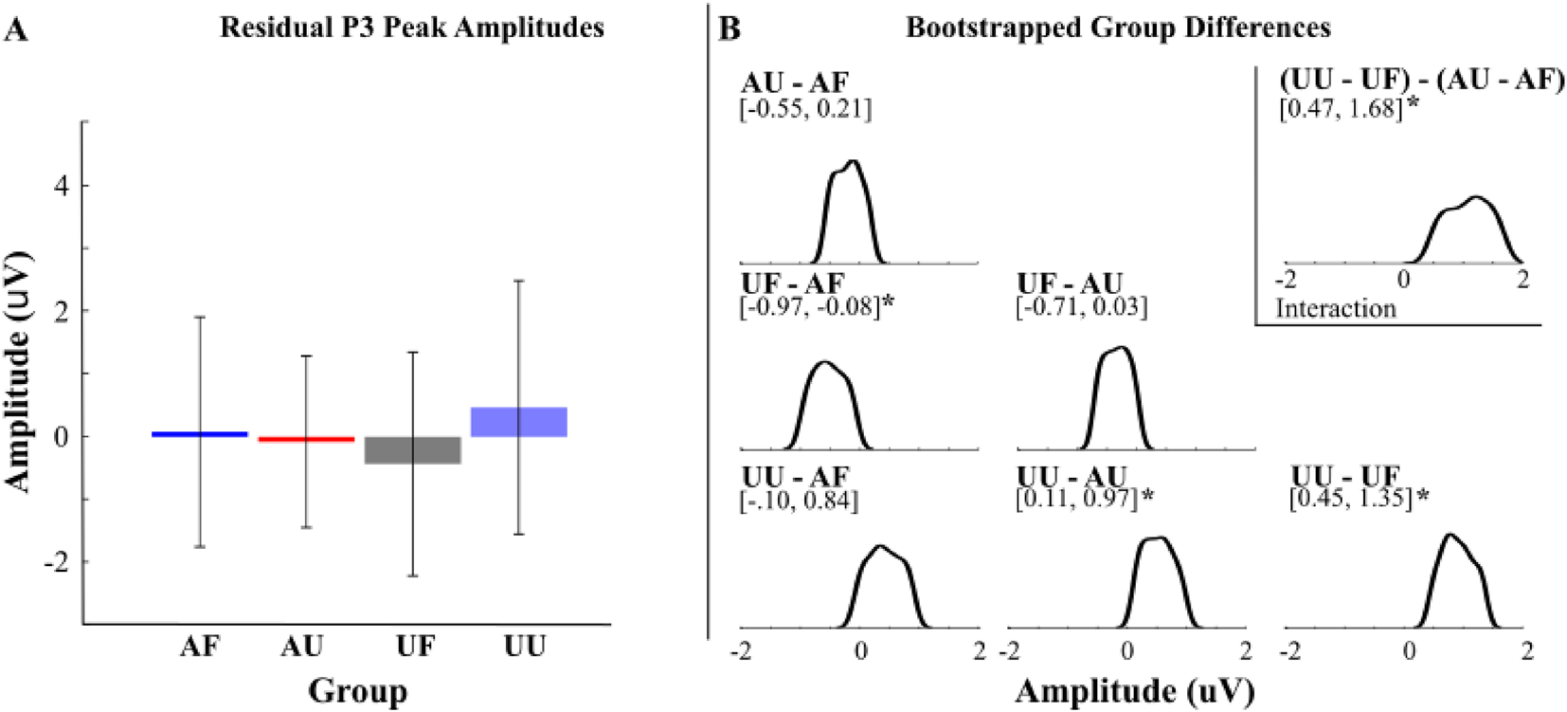
Residual P3 Amplitude. (**A**) Group average peak P3 residual amplitudes. (**B**) Distribution plots of group bootstrapped difference of paired comparisons. *p < 0.05.

Regarding participants’ neural responses to social exclusion, percentile bootstrap tests indicated larger P3 amplitudes to social exclusion in the UU group, compared to the AF and UF groups (*p*s < 0.05). In addition, the AF group had larger P3 amplitudes to social exclusion compared to the UF group (*p* < 0.05). Although P3 amplitudes were similar between the AF (i.e., most desirable qualities) and the UU (i.e., least desirable qualities), we observed an interaction between attractiveness and friendliness such that exclusion from unattractive women elicited a significantly larger P3 response when women were unfriendly compared to friendly (UU > UF), whereas P3 amplitudes were similar when being excluded by attractive women irrespective of their friendliness (Fig. 3). The group differences that were observed for raw P3 amplitudes remained unchanged after accounting for individual differences in rejection sensitivity (Fig. 4).

### Ratings of competitors’ attributes

Due to missing responses, for ratings of happy, nice, competitive, and attractive, participants included 79 women (AF = 19, AU = 21, UF = 20, UU = 19). Two additional participants in the UF condition did not provide one rating for rude (UF = 18).

### Happy

There was a significant main effect of competitor, *F*(2.89, 216.85) = 47.679, *p* < 0.001, partial eta-squared η_p_^2^ = 0.389, as well as an interaction between competitor and condition, *F*(8.67, 216.85) = 46.221, *p* < 0.001, η_p_^2^ = 0.649. Pair-wise comparisons indicated that in the attractive-friendly condition, UF2 was rated as less happy than all others and UF1 was rated less happy than AverageF and AF1. In the attractive unfriendly condition, the attractive unfriendly competitors were rated lower than others (AU1 < AU2 < UF2 < UF1 = AverageF). Participants in the unattractive friendly condition rated UF2 as lower than UF1, AF1, and AverageF. AF2 was also rated lower than AF1 and AverageF. In the unattractive unfriendly condition, UU1 and UU2 were rated as less happy than all other players (Fig. 3aS and Table 2S).

### Nice

There was a significant main effect of competitor, *F*(3.53, 265.04) = 17.492, *p* < 0.001, η_p_^2^ = 0.189, as well as an interaction between competitor and condition, *F*(10.60, 265.04) = 11.938, *p* < 0.001, η_p_^2^ = 0.323. Pair-wise comparisons indicated that in the attractive friendly condition, participants rated AF2 as less nice than AF1 and AverageF. In the attractive unfriendly condition, both attractive unfriendly competitors were rated as less nice than all other competitors. UF2 was in turn rated as less nice than AverageF. In the unattractive friendly condition, UF1 and UF2 were rated as less nice than AF1 and AverageF. In the unattractive unfriendly condition, UU1 and UU2 were rated as less nice than all other competitors (Fig. 3bS and Table 2S).

### Competitive

There was a significant main effect of competitor, *F*(5.54, 265.10) = 3.898, *p* = 0.006, η_p_^2^ = 0.049, as well as an interaction between competitor and condition, *F*(10.60, 265.10) = 5.284, *p* < 0.001, η_p_^2^ = 0.174. Pair-wise comparisons indicated that in the attractive friendly condition, AF1 and AF2 were rated more competitive than AverageF. AF2 was rated more competitive than UF1. In the attractive unfriendly condition, both attractive unfriendly competitors were rated as more competitive than UF1 and AverageF. In the unattractive friendly condition, UF1 was rated as more competitive than attractive competitors and when unattractive competitors were presented as unfriendly, they were rated as more competitive than all other competitors (Fig. 3cS and Table 2S).

### Rude

There was a significant main effect of competitor, *F*(2.73, 198.89) = 7.517, *p* < 0.001, η_p_^2^ = 0.093, as well as an interaction between competitor and condition, *F*(8.17, 198.89) = 5.797, *p* < 0.001, η_p_^2^ = 0.192. Pair-wise comparisons indicated that in the attractive friendly condtion, AF2 was rated as more rude than AF1, UF1, and AverageF. In addition, UF2 was rated as more rude than AverageF. When presented as unfriendly, attractive competitors were rated as more rude than AverageF but not unattractive competitors. When playing against unattractive competitors, participants rated them as more rude than attractive and average friendly competitors, with one exception—UF2 not different than AF2 (Fig. 3dS and Table 2S).

### Attractive

There was a significant main effect of competitor, *F*(2.33, 174.94) = 92.675, *p* < 0.001, η_p_^2^ = 0.553, as well as an interaction between competitor and condition, *F*(7.00, 174.94) = 4.081, *p* < 0.001, η_p_^2^ = 0.140. Pair-wise comparisons indicated that in the attractive friendly condition, attractive competitors were rated as more attractive than all other competitors and UF1 was rated as less attractive than UF2 and AverageF. In the attractive unfriendly condition, attractive unfriendly competitors were rated as more attractive than UF1. UF1 was rated as less attractive than AverageF. In the unattractive conditions, attractive competitors were rated as the most attractive. AverageF was rated as more attractive than UF1 and both unattractive unfriendly competitors. In the unattractive friendly condition, UF1 was rated as less attractive than UF2 (Fig. 3eS and Table 2S).

**Figure 5:**
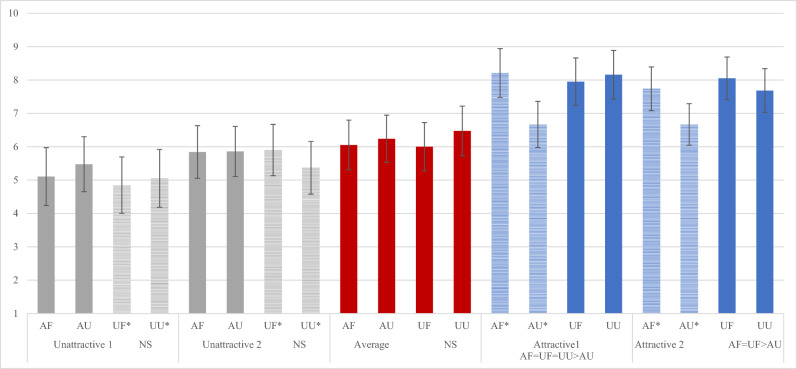
Ratings of attractiveness by photo. *opponent; U1= unattractive opponent 1; N= neutral non-opponent; A1= attractive opponent 1; U2= unattractive opponent 2; A2= attractive opponent 2.

When examining differences of attractiveness ratings across conditions within photographs, we found that there were no differences in attractiveness of the unattractive or average competitors between conditions; however, attractive competitor 1 was rated as less attractive by participants in the attractive unfriendly condition compared to all other conditions. Attractive competitor 2 was rated as less attractive by participants in the attractive unfriendly condition compared attractive friendly and unattractive friendly conditions (Fig. 5).

### Ratings of competitor attributes controlling for rejection sensitivity

When ratings of attributes by condition interactions were examined controlling for rejection sensitivity, all statistically significant attributes and pairwise comparison remained significant following the BH correction.

### Manipulation check

Participants in each of the conditions estimated the same number of throws *F*(3,79) = 0.136, *p* = 0.938, and reported similar levels of feeling ignored, *F*(3,79) = 0.868, *p* = 0.461, and excluded, *F*(3,75) = 1.490, *p* = 0.224. In response to being asked what they thought was the purpose of the study, only two participants thought that it was to assess how physical appearance or attractiveness was related to social exclusion, competing, or aggressive behavior (“Based on appearance, who you would be more competitive against or likely to throw the ball to” and “Whether there is a correlation between competitiveness and the way people look”). Most participants thought the study was about social exclusion (47.0%) and competitiveness (21.7%).

## Discussion

Using an evolutionary approach, we experimentally examined the behavioral and neural responses of social exclusion on women and, specifically, the role of facial attractiveness and friendliness, markers of social status. Our overarching prediction was that women would be most “hurt” by being excluded by attractive unfriendly (i.e., mean) women because these women could more readily inflict harm than women with lower social status^41^.

We found that women were more likely to select attractive women to compete against when all the potential competitors were presented as friendly and when the unattractive competitors were presented as unfriendly. When the attractive women were presented as unfriendly, the White attractive competitor was selected most often. This pattern of findings contradicts our a priori hypothesis. Attractive mean women have been shown to be a threat to other women^21,55^, and as such, we expected that they would be avoided as potential opponents. Our results, however, align with the “what is beautiful is good” stereotype in which attractive people are assumed to hold more positive personality traits and social qualities than less attractive people^87^. The observation that the attractive Black competitor was chosen less frequently than their attractive White counterpart under conditions where both were perceived as unfriendly could indicate the influence of stereotyping. Several studies have documented evidence of racial prejudice and the harmful trope of the “angry black woman” (e.g.,^88^), which may have influenced participants’ selections. However, it is also possible that this result was due to another unique, non-racial distinction.

We expected that women in the attractive unfriendly condition would report the highest level of ostracism-induced need threat and highest P3 amplitude. We found no differences by condition for level of ostracism, which is consistent with meta-analytic findings showing that the average effect for ostracism is large and robust across structural and sampling features, as well as different types of dependent measures^89^. Contrary to predictions, being rejected by attractive-friendly (i.e., most desirable qualities) and unattractive-unfriendly (i.e., least desirable qualities) elicited similar P3 amplitudes. Additionally, even after accounting for rejection sensitivity, there was an interaction between attractiveness and friendliness on P3 amplitudes such that exclusion from unattractive women elicited a significantly larger P3 response when the women were unfriendly compared to friendly (UU > UF), whereas P3 amplitudes were similar when being excluded by attractive women irrespective of their friendliness. It is possible that the higher P3 amplitude was due to an expectancy violation. Women may have expected to be treated poorly (i.e., excluded) by attractive women regardless of their level of friendliness based on experience, but they did not expect to be treated poorly by unattractive unfriendly opponents. Past research has shown that indirect aggression, which includes social exclusion, is more commonly employed by attractive women (and girls) than by less attractive women^21,55^. The idea that this may be an expectancy violation is consistent with earlier criticism of the Cyberball task which was purported to not measure “distressing experience of social rejection but rather was a by-product of the fact that the task involved an expectancy violation (being unexpectedly excluded)”^3^^,p.607^. More recent evidence, however, suggests that social response to exclusion is in fact present across different types of negative affect^3^.

The larger P3 in relation to being excluded by unattractive unfriendly women may be related to being offended by the rejection of an “inferior” opponent. Physical attractiveness influences access to social power, with less attractive individuals wielding less social power than more attractive individuals^17,41,90^. Studies examining social power (capacity to guide or influence the cognitive processes, emotional experiences, or observable actions of others) and ERP components have shown more enhanced P3 amplitudes in participants who were primed on social power^76,91^. The larger P3 amplitude observed in the present study might therefore reflect the greater attention salience engaged by the annoyance or disdain for being excluded by more socially subordinate women. Given that people tend to overestimate their own level of attractiveness (e.g.,^92,93^), especially unattractive people^94^, it is likely that participants perceived the unattractive unfriendly women to be less attractive than them, and thus their reaction was in line with “how dare she” or “who does she think she is?”. Given this unexpected finding, we examined post-hoc, anger rumination (assessed at the end of the study using Eken’s 6^95^ item measure (sample item: “It is going to annoy me for a while they did not throw to me.”; α = 0.885), the dwelling on perceived injustices, slights, or offenses^96^ in relation to exclusion condition. Because rejection sensitivity has been shown to predict increased rumination^97^, the interaction effect between condition and rejection sensitivity on anger rumination was assessed. Results indicated a statistically marginal association, *F*(3, 74) = 2.368, *p* = 0.078, which when probed, revealed that at high levels of rejection sensitivity, *F*(3,74) = 3.462, *p* = 0.021, but not at low or average levels, participants in the unattractive unfriendly condition reported higher anger rumination levels compared to other groups (UU = 2.38; AH = 1.44, *p* = 0.003; AU = 1.517, *p* = 0.008; UH = 1.659, *p* = 0.085). Thus, it seems that the increased P3 amplitude was likely a negative reaction to being excluded specifically by unattractive unfriendly women.

The ratings of competitors’ attributes provided evidence of validity for the experimental manipulation. Participants in the unfriendly conditions rated their opponents as being less happy and nice than participants in the friendly conditions, and across conditions, they tended to rate their opponents as being more competitive and more rude. Considering that participants were excluded by their opponents, these ratings make sense, and are consistent with studies showing that being socially excluded elicits retaliation^68,69^. Controlling for rejection sensitivity did not change the strength or direction of these findings, suggesting that the effects were robust.

Given our focus on the moderating role of attractiveness, we examined the participants’ ratings of attractiveness two ways—by condition and by photograph. By condition, participants’ ratings of opponents’ attractiveness were consistent with the ratings from the Chicago Face Database^76^. Across the four conditions, the attractive women were rated the highest on attractiveness, the unattractive women the lowest, and the average woman’s rating fell in the middle. By photograph, there were no differences found for the unattractive opponents as a function of friendliness (nor for the average women for whom they did not compete against), but there were differences for the attractive women by friendliness. Specifically, the attractiveness ratings for both attractive opponents were reduced in the unfriendly conditions. This result suggests that participants may have punished attractive unfriendly women when they were excluded by them. The punishment of attractive women by other women has been reported in previous studies. For example, Vaillancourt and Sharma^36^ found that almost all the women in their experimental study used indirect aggression against the attractive confederate when she was dressed in a sexually provocative manner. Muggleton et al.^32^ found that although men and women were negatively biased against sexually accessible women, it was women, and not men, who inflicted costly punishment upon them.

### Limitations

The present study has many strengths including its novelty, experimental design, racially/ethnically diverse sample, relatively large sample for an EEG study, careful attention to biases (i.e., inclusion of several manipulation checks and sensitivity analyses), and the use of ERP to confirm the attitude reports demonstrating that participants’ judgments were elicited quickly and were thus not just evaluations made in response to the questionnaires. Despite these strengths, there are limitations to consider. One, the relatively small sample sizes within each condition highlights the need for replication. Two, the limited racial and ethnic diversity of the photograph stimuli precluded an in-depth assessment of racial prejudice. It is challenging to incorporate racially and ethnically diverse photo stimuli into the Cyberball paradigm in a single study. Still, the absence of an unattractive Black comparison poses a limitation as it could have influenced participants' reaction to being socially excluded.

Three, despite good evidence and rationale for the sex differentiated aspects of social exclusion, including male photo stimuli and male participants would have provided an opportunity to test for sex differences in our study. We originally intended to conduct a second study of this nature; however, restricted variance in attractiveness ratings for the male faces in the Chicago Face Database dissuaded us from continuing. Therefore, in future research, it would be prudent to directly examine sex differences in response to social exclusion using the Cyberball paradigm via EEG with objective photo stimuli that have adequate variability in attractiveness ratings. This is an important avenue to explore given neuroimaging results indicating that being social excluded by one’s own sex is more distressing than being excluded by the opposite sex. Specifically, same-sex exclusion is associated with greater ventral anterior cingulate cortex activation, which is associated with the processing of negative emotions, notably sadness, and with self-reported feelings of distress in relation to social exclusion (e.g.,^98^).

Four, participants were all young women attending university; other social and age groups may respond differently. However, young women are more likely to be targets of indirect aggression than older women due to intrasexual competition^21^, and older adults tend to be less sensitive to social exclusion than younger adults^99–101^. Thus, if anything, our results are likely to be less pronounced in older women and enhanced in older adolescent girls.

Five, our focus on women centered around extant work showing that they engage in, are subjected to, and are harmed by social exclusion more than men in contemporary society^21,22^. Nevertheless, social affiliation and coalitional alliances are also important to men, and future work should seek to test these hypotheses in men. We expect that attractiveness will likely contribute less to men’s social positioning and that rival prestige or physical strength will be more relevant to how men respond to social exclusion^17^. In future studies it will also be important to examine the characteristics of socially excluded women and how they influence their appraisal and reaction to being rejected. For example, women interpret other women’s facial expressions of disgust as a cue of imminent social exclusion. This cue caused them harm, but especially so for women who were prone to being concerned with social belonging^23^.

In sum, we examined how women respond to social exclusion, with a focus on the impact of exclusion by either attractive or unattractive women, and their perceived level of friendliness. Although women have been shown to have “different default behavioral reactions to [social] threats” than men^22^^,p.543^, what has yet to be considered is how the characteristics of those imposing a social threat influence women’s reactions to social exclusion. In the present study, we found that women were most affected by being socially excluded by unattractive, unfriendly women. The reasons why are likely complex and multifaceted and require more investigation. Further investigation is needed because the use of indirect aggression, which includes social exclusion, is widespread among women and has profound emotional and physiological effects on their well-being^21^.

### Supplementary Information

Supplementary Information.

## Data Availability

The data that support the findings of this study are available upon request from the corresponding author.
