# Supplementary material for: Behavioral and neural responses to social exclusion in women: the role of facial attractiveness and friendliness
Source: Sci Rep. 2024 Jul 2;14:15135. doi: 10.1038/s41598-024-65833-4 (PMC11219880; doi:10.1038/s41598-024-65833-4)
Supplement: Supplementary file 1 — Supplementary Information. [file 41598_2024_65833_MOESM1_ESM.docx]

| Supplemental Figure 1. *Schematic Overview of the EEG Pre-Processing Sequence* | | | |
| --- | --- | --- | --- |
|  | **Criteria threshold** | **Outlier Flag** | **%** |
| **Scalp signal quality assessment** | | | |
| Co-registration to reference head surface |  |  |  |
| Variance time point criteria  Variance channel criteria | 3x > .15-.85 quantile range  3x > .15-.85 quantile range | > 20% of channels  > 20% of epochs | 0  6.2 |
| Interpolated average reference  High pass filter (1 Hz)  Low pass filter (30 Hz) |  |  |  |
| Correlation channel criteria  Bridge channel criteria  Rank channel selection | 3x > .15-.85 quantile range  Max *r* *M/SD* > 6 *SD* of 40% trim mean | > 20% of epochs | 4.1 |
| Interpolated average reference |  |  |  |
| Correlation time period criteria | 3x > .15-.85 quantile range | > 20% of channels | 1.8 |
| **Initial ICA signal quality assessment** | | | |
| IC variance time point criteria | 3x > .15-.85 quantile range | > 20% of components | 2.8 |
| **Final ICA signal quality assessment** | | | |
| IC variance time period criteria  IC dipole fit  IC label component classification | 3x > .15-.85 quantile range | > 20% of components | 0.16 |
| **Interactive quality control assessment** | | | |
| Visual inspection of artifacts |  | | |
| **Signal purge and data reduction** | | | |
| *Legend.* Signal Quality Assessment Unit: Data manipulation □; Automatic annotation criteria □; Interactive inspection □ | | | |

Supplemental Figure 2*.* *Analytic Plan Flowchart*

Prediction 3: Being socially excluded by attractive unfriendly women will elicit the largest P3 response.

Sensitivity Analysis: Controlling for rejection sensitivity

McNemar test of proportions

General linear model

Percentile Bootstrap

Repeated measures ANOVA

Repeated measures ANCOVA

One-way ANOVA

Prediction 1: Women will favor playing against attractive unfriendly women the least and unattractive friendly women the most.

Validity Analyses

Prediction 4: Women will rate their competitors as being more rude, more competitive, less attractive, less nice, and less happy than their non-competitors.

Sensitivity Analysis: Controlling for rejection sensitivity

Prediction 2: Being socially excluded by attractive unfriendly women will elicit the strongest feelings of ostracism.

Supplemental Figure 3a. *Ratings of happy by condition*

*opponent; U1= unattractive opponent 1; U2= unattractive opponent 2; N= neutral non-opponent; A1= attractive opponent 1; A2= attractive opponent 2

Supplemental Figure 3b. *Ratings of nice by condition*

*opponent; U1= unattractive opponent 1; U2= unattractive opponent 2; N= neutral non-opponent; A1= attractive opponent 1; A2= attractive opponent 2

Supplemental Figure 3c. *Ratings of competitive by condition*

*opponent; U1= unattractive opponent 1; U2= unattractive opponent 2; N= neutral non-opponent; A1= attractive opponent 1; A2= attractive opponent 2

Supplemental Figure 3d. *Ratings of rude by condition*

*opponent; U1= unattractive opponent 1; U2= unattractive opponent 2; N= neutral non-opponent; A1= attractive opponent 1; A2= attractive opponent 2

Supplemental Figure 3e. *Ratings of attractiveness by condition*

*opponent; U1= unattractive opponent 1; U2= unattractive opponent 2; N= neutral non-opponent; A1= attractive opponent 1; A2= attractive opponent 2

| Supplemental Table 1. *Statistical Summary of Opponent Attributes by Condition* | | | | | |
| --- | --- | --- | --- | --- | --- |
| **Condition and Post-Hoc Comparisons** | **Photo** | **Mean** | **Std. Error** | **95% Confidence Interval** | |
|  |  |  |  | ***Lower Bound*** | ***Upper Bound*** |
| Happy | | | | | |
| Attractive Friendly  U2<U1,A2,Average,A1; U1<Average,A1 | U1 | 7.526 | 0.388 | 6.754 | 8.299 |
|  | U2 | 6.421 | 0.399 | 5.625 | 7.217 |
|  | Average | 8.474 | 0.290 | 7.895 | 9.052 |
|  | A1* | 8.579 | 0.342 | 7.898 | 9.260 |
|  | A2* | 7.947 | 0.403 | 7.144 | 8.751 |
| Attractive Unfriendly  A1<A2<U2<U1=Average | U1 | 8.762 | 0.369 | 8.027 | 9.497 |
|  | U2 | 7.667 | 0.380 | 6.910 | 8.424 |
|  | Average | 9.190 | 0.276 | 8.640 | 9.741 |
|  | A1* | 4.381 | 0.325 | 3.733 | 5.029 |
|  | A2* | 5.429 | 0.383 | 4.665 | 6.193 |
| Unattractive Friendly  U2<U1,A1,Average; A2<A1,Average | U1* | 8.000 | 0.378 | 7.247 | 8.753 |
|  | U2* | 7.050 | 0.389 | 6.274 | 7.826 |
|  | Average | 8.550 | 0.283 | 7.986 | 9.114 |
|  | A1 | 8.550 | 0.333 | 7.886 | 9.214 |
|  | A2 | 7.600 | 0.393 | 6.817 | 8.383 |
| Unattractive Unfriendly  U1=U2<Average,A1,A2 | U1* | 3.737 | 0.388 | 2.964 | 4.509 |
|  | U2* | 3.789 | 0.399 | 2.994 | 4.585 |
|  | Average | 8.789 | 0.290 | 8.211 | 9.368 |
|  | A1 | 9.000 | 0.342 | 8.319 | 9.681 |
|  | A2 | 8.526 | 0.403 | 7.723 | 9.329 |
| Nice | | | | | |
| Attractive Friendly  A2<Average,A1 | U1 | 7.211 | 0.414 | 6.386 | 8.035 |
|  | U2 | 7.000 | 0.446 | 6.112 | 7.888 |
|  | Average | 7.895 | 0.358 | 7.182 | 8.607 |
|  | A1* | 7.684 | 0.416 | 6.855 | 8.513 |
|  | A2* | 6.421 | 0.458 | 5.509 | 7.334 |
| Attractive Unfriendly  A1=A2<U2<Average; A1=A2<U1 | U1 | 8.095 | 0.394 | 7.311 | 8.880 |
|  | U2 | 7.238 | 0.424 | 6.393 | 8.083 |
|  | Average | 8.381 | 0.340 | 7.703 | 9.059 |
|  | A1* | 5.810 | 0.396 | 5.021 | 6.598 |
|  | A2* | 5.810 | 0.436 | 4.942 | 6.678 |
| Unattractive Friendly  U1<A1,Average; U2<Average,A1 | U1* | 6.300 | 0.404 | 5.496 | 7.104 |
|  | U2* | 6.500 | 0.435 | 5.634 | 7.366 |
|  | Average | 7.900 | 0.349 | 7.206 | 8.594 |
|  | A1 | 7.850 | 0.405 | 7.042 | 8.658 |
|  | A2 | 7.300 | 0.446 | 6.411 | 8.189 |
| Unattractive Unfriendly  U1=U2<Average,A1,A2 | U1* | 5.211 | 0.414 | 4.386 | 6.035 |
|  | U2* | 4.421 | 0.446 | 3.533 | 5.310 |
|  | Average | 8.263 | 0.358 | 7.551 | 8.976 |
|  | A1 | 8.526 | 0.416 | 7.698 | 9.355 |
|  | A2 | 8.053 | 0.458 | 7.140 | 8.965 |
| Competitive |  |  |  |  |  |
| Attractive Friendly  A2>U1,Average; A1>Average | U1 | 3.789 | 0.592 | 2.611 | 4.968 |
|  | U2 | 4.158 | 0.607 | 2.949 | 5.366 |
|  | Average | 3.263 | 0.582 | 2.104 | 4.422 |
|  | A1* | 5.053 | 0.487 | 4.083 | 6.023 |
|  | A2* | 5.632 | 0.559 | 4.518 | 6.745 |
| Attractive Unfriendly  A1=A2>U1,Average | U1 | 3.381 | 0.563 | 2.260 | 4.502 |
|  | U2 | 3.905 | 0.577 | 2.755 | 5.054 |
|  | Average | 3.619 | 0.553 | 2.517 | 4.721 |
|  | A1* | 4.952 | 0.463 | 4.030 | 5.875 |
|  | A2* | 4.905 | 0.532 | 3.846 | 5.964 |
| Unattractive Friendly  U1>A1,A2 | U1* | 5.100 | 0.577 | 3.951 | 6.249 |
|  | U2* | 4.450 | 0.591 | 3.272 | 5.628 |
|  | Average | 4.000 | 0.567 | 2.870 | 5.130 |
|  | A1 | 3.500 | 0.475 | 2.555 | 4.445 |
|  | A2 | 3.450 | 0.545 | 2.365 | 4.535 |
| Unattractive Unfriendly  U1=U2>Average,A1,A2 | U1* | 6.053 | 0.592 | 4.874 | 7.231 |
|  | U2* | 6.211 | 0.607 | 5.002 | 7.419 |
|  | Average | 3.737 | 0.582 | 2.578 | 4.896 |
|  | A1 | 4.053 | 0.487 | 3.083 | 5.023 |
|  | A2 | 4.000 | 0.559 | 2.887 | 5.113 |
| Rude |  |  |  |  |  |
| Attractive Friendly  A2>U1,Average,A1; U2>Average | U1 | 2.105 | 0.467 | 1.175 | 3.035 |
|  | U2 | 3.105 | 0.522 | 2.065 | 4.146 |
|  | Average | 1.947 | 0.351 | 1.248 | 2.646 |
|  | A1* | 2.474 | 0.370 | 1.736 | 3.211 |
|  | A2* | 3.842 | 0.495 | 2.855 | 4.829 |
| Attractive Unfriendly  A1,A2>Average | U1 | 2.000 | 0.444 | 1.115 | 2.885 |
|  | U2 | 2.000 | 0.497 | 1.010 | 2.990 |
|  | Average | 1.905 | 0.334 | 1.240 | 2.570 |
|  | A1* | 3.095 | 0.352 | 2.394 | 3.797 |
|  | A2* | 3.048 | 0.471 | 2.108 | 3.987 |
| Unattractive Friendly  U1>Average,A1,A2; U2>Average,A1 | U1* | 4.167 | 0.479 | 3.211 | 5.122 |
|  | U2* | 3.278 | 0.536 | 2.209 | 4.347 |
|  | Average | 2.056 | 0.360 | 1.337 | 2.774 |
|  | A1 | 1.889 | 0.380 | 1.131 | 2.647 |
|  | A2 | 2.333 | 0.509 | 1.319 | 3.348 |
| Unattractive Unfriendly  U1=U2>Average,A1,A2 | U1* | 3.895 | 0.467 | 2.965 | 4.825 |
|  | U2* | 4.158 | 0.522 | 3.118 | 5.198 |
|  | Average | 1.947 | 0.351 | 1.248 | 2.646 |
|  | A1 | 1.737 | 0.370 | 0.999 | 2.474 |
|  | A2 | 1.947 | 0.495 | 0.960 | 2.935 |
| Attractive |  |  |  |  |  |
| Attractive Friendly  A1=A2>U2=Average>U1 | U1 | 5.105 | 0.442 | 4.225 | 5.986 |
|  | U2 | 5.842 | 0.403 | 5.039 | 6.645 |
|  | Average | 6.053 | 0.380 | 5.296 | 6.809 |
|  | A1* | 8.211 | 0.372 | 7.469 | 8.952 |
|  | A2* | 7.737 | 0.334 | 7.071 | 8.403 |
| Attractive Unfriendly  A1=A2>U1; Average>U1 | U1 | 5.476 | 0.421 | 4.638 | 6.314 |
|  | U2 | 5.857 | 0.383 | 5.094 | 6.621 |
|  | Average | 6.238 | 0.361 | 5.519 | 6.958 |
|  | A1* | 6.667 | 0.354 | 5.962 | 7.372 |
|  | A2* | 6.667 | 0.318 | 6.033 | 7.300 |
| Unattractive Friendly  A1=A2>Average=U2>U1 | U1* | 4.850 | 0.431 | 3.992 | 5.708 |
|  | U2* | 5.900 | 0.393 | 5.118 | 6.682 |
|  | Average | 6.000 | 0.370 | 5.263 | 6.737 |
|  | A1 | 7.950 | 0.363 | 7.228 | 8.672 |
|  | A2 | 8.050 | 0.326 | 7.401 | 8.699 |
| Unattractive Unfriendly  A1=A2>Average>U1=U2 | U1* | 5.053 | 0.442 | 4.172 | 5.933 |
|  | U2* | 5.368 | 0.403 | 4.566 | 6.171 |
|  | Average | 6.474 | 0.380 | 5.717 | 7.230 |
|  | A1 | 8.158 | 0.372 | 7.417 | 8.899 |
|  | A2 | 7.684 | 0.334 | 7.018 | 8.350 |
| U1 = unattractive photo 1; U2 = unattractive photo 2; A1 = attractive photo 1; A2 = attractive photo 2 | | | | | |
